# Pemetrexed plus Platinum as the First-Line Treatment Option for Advanced Non-Small Cell Lung Cancer: A Meta-Analysis of Randomized Controlled Trials

**DOI:** 10.1371/journal.pone.0037229

**Published:** 2012-05-17

**Authors:** Ming Li, Qian Zhang, Peifang Fu, Ping Li, Aimei Peng, Guoliang Zhang, Xiaolian Song, Min Tan, Xuan Li, Yang Liu, Yueping Wu, Suyun Fan, Changhui Wang

**Affiliations:** 1 Department of Respiratory Medicine, Shanghai Tenth People's Hospital, Tongji University, Shanghai, China; 2 Division of Nephrology, Huashan Hospital, Fudan University, Shanghai, China; Faculté de médecine de Nantes, France

## Abstract

To compare the efficacy and toxicities of pemetrexed plus platinum with other platinum regimens in patients with previously untreated advanced non-small cell lung cancer (NSCLC). Methods: A meta-analysis was performed using trials identified through PubMed, EMBASE, and Cochrane databases. Two investigators independently assessed the quality of the trials and extracted data. The outcomes included overall survival (OS), progression-free survival (PFS), response rate (RR), and different types of toxicity. Hazard ratios (HRs), odds ratios (ORs) and their 95% confidence intervals (CIs) were pooled using RevMan software. Results: Four trials involving 2,518 patients with previously untreated advanced NSCLC met the inclusion criteria. Pemetrexed plus platinum chemotherapy (PPC) improved survival compared with other platinum-based regimens (PBR) in patients with advanced NSCLC (HR = 0.91, 95% CI: 0.83–1.00, *p* = 0.04), especially in those with non-squamous histology (HR = 0.87, 95% CI: 0.77–0.98, *p* = 0.02). No statistically significant improvement in either PFS or RR was found in PPC group as compared with PBR group (HR = 1.03, 95% CI: 0.94–1.13, *p* = 0.57; OR = 1.15, 95% CI: 0.95–1.39, *p* = 0.15, respectively). Compared with PBR, PPC led to less grade 3–4 neutropenia and leukopenia but more grade 3–4 nausea. However, hematological toxicity analysis revealed significant heterogeneities. Conclusion: Our results suggest that PPC in the first-line setting leads to a significant survival advantage with acceptable toxicities for advanced NSCLC patients, especially those with non-squamous histology, as compared with other PRB. PPC could be considered as the first-line treatment option for advanced NSCLC patients, especially those with non-squamous histology.

## Introduction

Lung cancer is the leading cause of cancer-related mortality in both men and women, resulting in approximately 221,130 new cases and 156,940 deaths within the United States in 2011 [1]. Lung cancer causes approximately 1.3 million deaths per year worldwide, and non-small cell lung cancer (NSCLC) represents 85% of all lung cancers. The 5-year survival of patients with metastatic NSCLC is less than 10% [2], [3]. Platinum-based doublet chemotherapy is the current standard of care for patients with preserved functional status. Patients treated with platinum-based regimens have a mean survival of 8–10 months. Despite advances in the treatment of advanced NSCLC, the advent of third-generation cytotoxic agents including gemcitabine and docetaxel has reached a therapeutic plateau [4].

Pemetrexed is a multi-targeted inhibitor of three key enzymes in the folate metabolic pathway: thymidylate synthase (TS), dihydrofolate reductase (DHFR) and glycinamide ribonucleotide formyl transferase (GARFT) [5], [6]. In 2008, Scagliotti et al. [7] compared first-line pemetrexed/cisplatin (PP) to gemcitabine/cisplatin (GP) and found that pemetrexed was not inferior in terms of overall survival (OS) (hazard ratio [HR] = 0.94, 95% confidence interval [CI]: 0.84–1.05). However, the result of subgroup analysis showed that pemetrexed improved OS in patients with non-squamous histology (adenocarcinoma and large cell carcinoma) (HR = 0.81, 95% CI: 0.70–0.94). Therefore, the pemetrexed-cisplatin regimen is recommended by the National Comprehensive Cancer Network (NCCN) guidelines as the first-line treatment for patients with advanced non-squamous NSCLC [8].

However, in 2009, Gronberg et al [9]. found that pemetrexed/carboplatin (PC) provided similar OS when compared with gemcitabine/carboplatin (GC), and that there was also no difference in OS when analyzing patients with non-squamous histology (7.8 months versus 7.5 months, *p* = 0.77). Another study [10] concluded that PC treatment was associated with significantly longer OS when compared with docetaxel/carboplatin (DC) (12.7 months versus 9.2 months, *p* = 0.05). Hence, the role of the pemetrexed plus platinum chemotherapy (PPC) in the treatment of advanced NSCLC remains undefined. The objective of this meta-analysis was to compare the efficacy and toxicities of PPC with other platinum-based regimens (PBR) in the treatment of patients with previously untreated advanced NSCLC.

## Methods

### Literature Search

An electronic sensitive search of PubMed, EMBASE and CENTRAL (the Cochrane Central Register of Controlled Trials) database was performed in December 2011, using the following key words as the search terms: “NSCLC”, “non-small cell lung cancer”, “pemetrexed or *Alimta or LY231514*”, “first-line”, and “chemotherapy-naive”. Only randomized controlled trials that fulfilled the criteria of a highly sensitive filter were included in this study [11]. The published languages and years were not limited. The relevant reviews and meta-analyses regarding the role of the first-line treatment for patients with NSCLC were examined for potential inclusive trials. References of all randomized clinical trials were scanned for additional study. The American Society of Clinical Oncology (ASCO) and European Society for Medical Oncology (ESMO) annual meeting abstracts in the latest 15 years were also searched.

### Selection Criteria

Trials were excluded if they did not meet with the below inclusion criteria. Trials were included if: (1) they compared PPC (pemetrexed plus cisplatin or carboplatin chemotherapy) with other PBR (third-generation agents plus cisplatin or carboplatin regimens); (2) enrolled NSCLC patients were previously untreated; and (3) treated patients had stage IIIB or IV NSCLC, regardless of the publication status (published, conference proceedings, or unpublished). Two investigators (L.M. and Z.Q.) independently inspected each reference and applied the inclusion criteria. For possibly relevant articles or in cases of disagreement, all investigators inspected the full text independently.

### Data Extraction And Quality Assessment

The two investigators independently extracted data from all primary studies that fulfilled the inclusion criteria, and any disagreement was resolved by consensus. In articles where outcomes were not reported, attempts were made to contact the authors for additional information. The following data were abstracted from each article with a standardized approach, including publication details, quality scores, trial characteristics (such as the number of the patients, chemotherapy regimens, age, gender, stage, and pathologic type), outcome measures (such as response rates [RR], HRs for OS and progression-free survival [PFS] and their 95% CIs, log-rank test *p* values), and specific grade 3–4 adverse events including hematological and nonhematological toxicities.

The same reviewers independently assessed trials for methodological quality by the Jadad scale [12], and any disagreement was resolved by consensus. The Jadad score was based on the explicit description of the study in the text as “randomized” and “double-blind”, and reporting of “withdrawals and dropouts”

### Statistical Analysis

Data were analyzed using Review Manager (RevMan, Version 5.0, Copenhagen: The Nordic Cochrane Centre, The Cochrane Collaboration, 2008). Time-to-event data were summarized by the log HR and its variance using previously reported methods [13]. Results were presented as HRs and 95% CIs using a general variance-based method. Dichotomous data were compared using an odds ratio (OR). Respective 95% CI was calculated for each estimate and presented in forest plots.

Statistical heterogeneity of the trial results was assessed with the *χ* ^2^ test for heterogeneity and the *I* ^2^ test for inconsistency [14]. If the *p* value was less than 0.1 (*χ* ^2^ test), the results were considered heterogeneous; if the *I* ^2^ was greater than 50%, the results were considered inconsistent [15]. If the test results for heterogeneity were significant, the DerSimonian and Laird random-effects model was used to analyze the treatment groups. The potential presence of publication bias was evaluated visually by inspecting funnel plots and statistically by the Egger's test [16].

## Results

### Search Of The Published Literature

**Figure 1:**
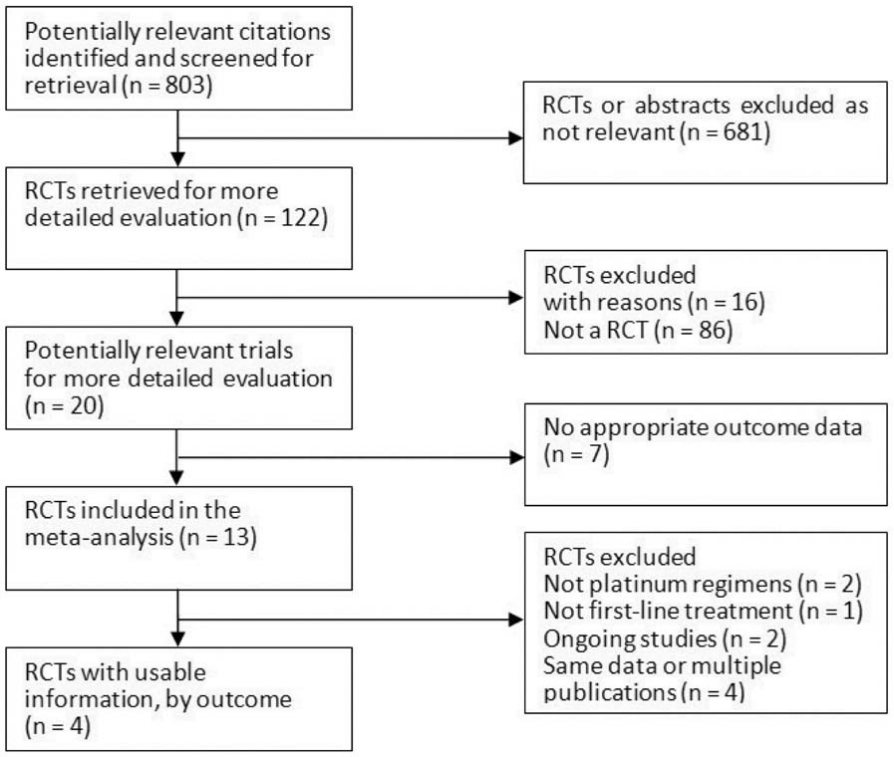
Procedures used for trial selection. Abbreviations: RCT, randomized controlled trial.

The literature search identified 803 publications on pemetrexed, 13 of which were potentially eligible trials that examined pemetrexed therapy in advanced NSCLC patients. Figure 1 shows the reasons for excluding 9 of these reports. Ultimately, four trials were included, all of which were performed between 2008 and 2011, involving a total of 2,518 advanced NSCLC patients [7], [9], [10], [17]. None of the conference abstracts met the inclusion criteria and therefore were not included for analysis. Multiple publications were excluded from the count of included studies because they were secondary publications of previous reports, though any relevant and unique results were extracted and included. The PRISMA Checklist and Flow Diagram for the studies is shown in Checklist S1 and Figure S1.

### Characteristics Of The Included Studies

The quality of the four trials was assessed with the three-question instrument proposed by Jadad et al. [12]. All the four trials included statements regarding randomization, and three of the trials described the detailed methods used for randomization [7], [9], [17]. Thus, all trials were scored as 1 or 2 based on randomization criteria. All trials reported withdrawals and drop-outs, but none of them specified the use of double-blind methodology. Three of the four trials were phase III RCTs [7], [9], [17], and the other trial [10] was phase II RCT. Only one trial [7] used cisplatin, and the others used carboplatin [9], [10], [17]. Two trials compared pemetrexed to gemcitabine [7], [9], and the other two trials compared pemetrexed to docetaxel [10], [17]. One of the four trials was a three-arm trial [10]. All the four trials were reported in full text. The baseline characteristics of the four trials are listed in Table 1.

**Table 1:** Characteristics of Studies Included in the Meta-analysis.

Study	Quality (Scores)	Therapy	n	Age Median	Male (%)	Stage IIIB(%)	Stage IV(%)	Non-squ (%)	OS Median	PFS Median
Scagliotti et al. [7]	3	PEM- 500 mg/m2 d1+P-75 mg/m2 d1, q3w	862	61.1	70.2	23.8	76.2	71.7	10.3	4.8
		GEM-1,250 mg/m2 d1,8+P-75 mg/m2 d1, q3w	863	61.0	70.1	24.3	75.7	73.5	10.3	5.1
Gronberg et al. [9]	3	PEM- 500 mg/m2 d1+P#-AUC 5 d1, q3w	219	64	56	29	71	74	7.3	NA
		GEM-1,000 mg/m2 d1,8+P#-AUC 5 d1, q3w	217	66	59	28	72	77	7.0	NA
Socinski et al. [10]	2	PEM- 500 mg/m2 d1+P#-AUC 6 d1, q3w	74	66	55	7	93	70	12.7	NA
		Doc-75 mg/m2 d1+P#-AUC 6 d1, q3w	72	65	58	8	92	81	9.2	NA
Rodrigues-Pereira et al. [17]	3	PEM- 500 mg/m2 d1+P#-AUC 5 d1, q3w	106	60.1	60.4	16	84	100	14.9	5.8
		Doc-75 mg/m2 d1+P#-AUC 5 d1, q3w	105	58.9	47.6	21.9	78.1	100	14.7	6.0

Abbreviations: PEM, pemetrexed; GEM, gemcitabine; Doc, docetaxel; P, cisplatin; P#, carboplatin; Ade, adenocarcinoma; Non-squ, non-squamous cell carcinoma; AUC, area under the concentration/time curve. NA, not available; OS, overall survival; progression-free survival.

### Overall Survival

**Figure 2:**
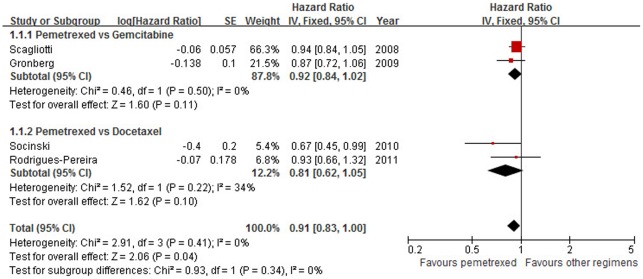
Comparison of overall survival between pemetrexed plus platinum chemotherapy and other platinum-based regimens. Abbreviations: SE, standard error; IV, inverse variance; CI, confidence interval.

All the four trials (comprising 2,518 cases) reported HRs for OS. Taken together, the HR for OS favored PPC (HR = 0.91, 95% CI: 0.83–1.00, *p* = 0.04), without evidence of heterogeneity between the studies (*I* ^2^ = 0%; *p* = 0.41) (Figure 2). The pooled HR for OS was performed using the fixed-effort model. The result indicates that PPC resulted in a slight but significant reduction in the risk of death (9%) compared with other PBR in advanced NSCLC. In addition, no publication bias was detected by Egger's test (*p* = 0.27).

Subgroup analysis was conducted according to the different drugs used in PBR. Compared with gemcitabine or docetaxel plus platinum, PPC showed a beneficial trend in terms of OS despite a lack of statistical significance (HR = 0.92, 95% CI: 0.84–1.02, *p* = 0.11; HR = 0.81, 95% CI: 0.62–1.05, *p* = 0.10, respectively) (Figure 2). There was no evidence of heterogeneity between the studies (*I* ^2^ = 0%, *p* = 0.50; *I* ^2^ = 34%, *p* = 0.22, respectively). There was no evidence of statistical interaction between the two subgroups (*p* = 0.36).

Sensitivity analysis was performed after the trial [7] using carboplatin was excluded because of the possible difference in efficacy between platinum agents. The result confirmed the benefit of PPC (HR = 0.85, 95% CI: 0.72–0.99, *p* = 0.04), with no evidence of heterogeneity (*I* ^2^ = 0%; *p* = 0.42).

**Figure 3:**
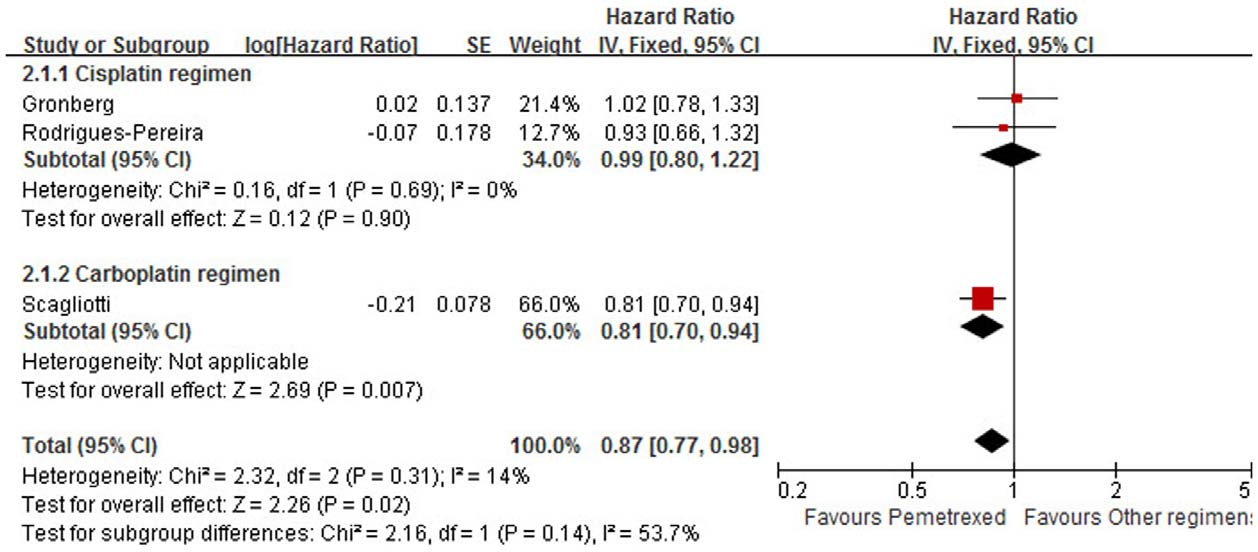
Comparison of overall survival in patients with nonsquamous histology between pemetrexed plus platinum chemotherapy and other platinum-based regimens. Abbreviations: SE, standard error; IV, inverse variance; CI, confidence interval.

Three trials [7], [9], [17] reported HRs for OS in patients with non-squamous histology (comprising 1,792 cases). Taken together, PPC was associated with a clinically and statistically significant 13% improvement in OS compared with other PBR (HR = 0.87, 95% CI: 0.77–0.98, *p* = 0.02), with no evidence of heterogeneity (*I* ^2^ = 14%; *p* = 0.31). Sensitivity analysis excluding the trial using carboplatin [7] did not confirm the above result (HR = 0.99, 95% CI: 0.80–1.22, *p* = 0.90), with no evidence of heterogeneity (*I* ^2^ = 0%; *p* = 0.69) (Figure 3). Subgroup and sensitivity analyses excluding the trial using docetaxel [17] also gave a negative result (HR = 0.88, 95% CI: 0.71–1.10, *p* = 0.27), with significant heterogeneity (*I* ^2^ = 53%; *p* = 0.14).

### Progression-free Survival

**Figure 4:**
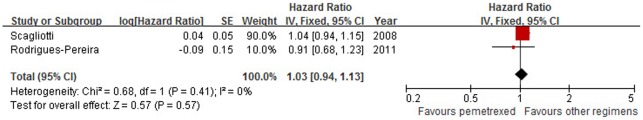
Comparison of progression-free survival between pemetrexed plus platinum chemotherapy and other platinum-based regimens. Abbreviations: SE, standard error; IV, inverse variance; CI, confidence interval.

Two trials [7], [17] reported HRs for PFS (comprising 1,936 cases). Compared with other PBR, PFS was not significantly better in patients who received PPC (HR = 1.03, 95% CI: 0.94–1.13, *p* = 0.57). There was no evidence of heterogeneity between the studies (*I* ^2^ = 0%, *p* = 0.41) (Figure 4).

### Overall Response Rates

**Figure 5:**
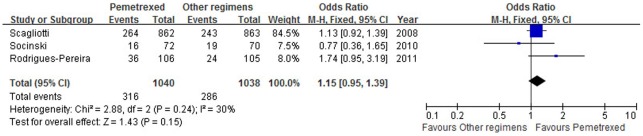
Comparison of response rate between pemetrexed plus platinum chemotherapy and other platinum-based regimens. Abbreviations: M-H, mantel-haenszel; CI, confidence interval.

Socinski et al. [10] reported both complete and partial responses. Three trials [7], [10], [17] reported overall response. Compared with other PBR, PPC showed a beneficial trend in terms of RR despite a lack of statistical significance (OR = 1.15, 95% CI: 0.95–1.39, *p* = 0.15). There was no evidence of heterogeneity between the studies (*I* ^2^ = 30%, *p* = 0.24) (Figure 5).

### Toxicity

#### Hematological Toxicity

**Figure 6:**
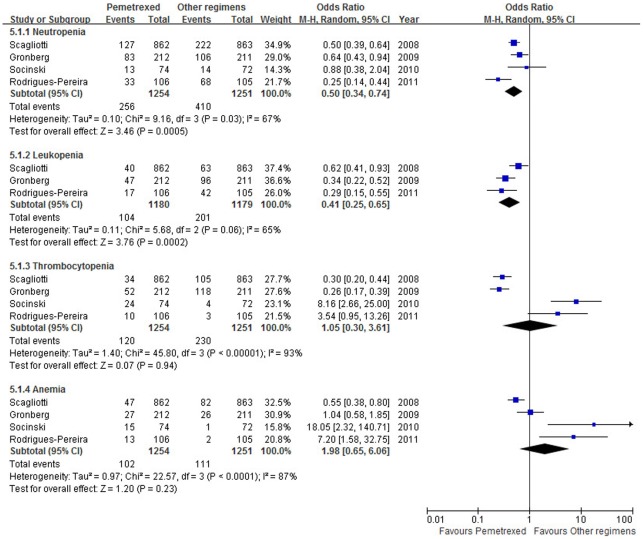
Summary of grade 3–4 hematological toxicity. Abbreviations: M-H, mantel-haenszel; CI, confidence interval.

Chemotherapy toxicity was described as patients experiencing grade 3–4 toxicity. Figure 6 is a summary of grade 3–4 hematological toxicity. All the four trials reported hematological toxicity, including neutropenia, anemia and thrombocytopenia. Only three trials [7], [9], [17] reported leukopenia. Compared with other PBR, PPC led to less grade 3–4 neutropenia and leukopenia (OR = 0.50, 95% CI: 0.34–0.74, *p* = 0.0005; OR = 0.41, 95% CI: 0.25–0.65, *p* = 0.0002, respectively). Compared with the gemcitabine-based regimen, a statistically significant decrease in thrombocytopenia but not in anemia was observed (OR = 0.28, 95% CI: 0.21–0.37, *p*<0.00001; OR = 0.72, 95% CI: 0.39–1.34, *p* = 0.30, respectively). Compared with the docetaxel-based regimen, a statistically significant increase in thrombocytopenia and anemia was observed (OR = 5.75, 95% CI: 2.45–13.52, *p*<0.0001; OR = 9.95, 95% CI: 2.94–33.68, *p* = 0.0002, respectively). The pooled ORs for hematological toxicity were performed using the random-effort model because of heterogeneities.

### Non-hematological Toxicity

**Figure 7:**
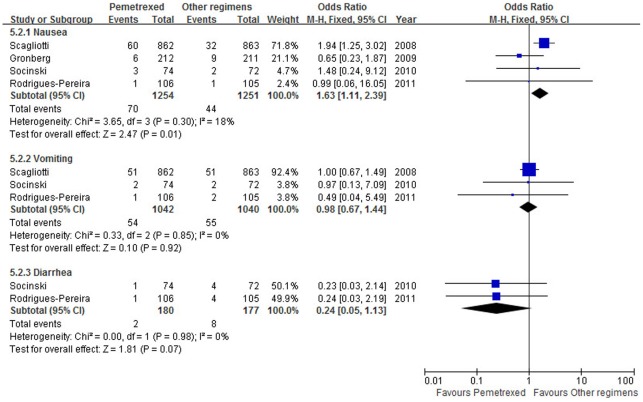
Summary of grade 3–4 nonhematological toxicity. Abbreviations: M-H, mantel-haenszel; CI, confidence interval.

Figure 7 is a summary of grade 3–4 non-hematological toxicity. All the four trials reported nausea, three trials [7], [10], [17] reported vomiting, and two trials [10], [17] reported diarrhea. Compared with other PBR, PPC led to more grade 3–4 nausea (OR = 1.63, 95% CI: 1.11–2.39, *p* = 0.01) but not vomiting and diarrhea (OR = 0.98, 95% CI: 0.67–1.44, *p* = 0.92; OR = 0.24, 95% CI: 0.05–1.13, *p* = 0.07, respectively). There was no significant heterogeneity for all the nonhematological toxicity analyses.

## Discussion

The current standard first-line treatment for patients with advanced NSCLC is platinum-based doublets with third-generation agents (i.e. gemcitabine, paclitaxel, docetaxel, irinotecan and vinorelbine). A previous meta-analysis [18] by Grossi et al. found comparable activity between the third-generation regimens in the first-line treatment of advanced NSCLC. Pemetrexed is a novel multi-targeted antifolate chemotherapy agent that primarily inhibits TS. In 2008, Scagliotti et al. [7] first reported a large phase III study to compare PP with GP, finding that pemetrexed significantly improved OS in non-squamous patients but significantly decreased OS in squamous patients. Based on this study, pemetrexed has been granted as the first-line treatment for patients with advanced non-squamous NSCLC. In 2009, Gronberg et al. [9] reported another phase III study, but they did not demonstrate any significant association between histology and survival. Recently, Rodrigues-Pereira et al. [17] also reported a negative result. Therefore, we preformed a meta-analysis to evaluate the efficacy and safety of PPC as the first-line chemotherapy in patients with advanced NSCLC.

The main finding of the present meta-analysis is that PPC improved OS homogenously and significantly, when compared with other PBR, with a 9% reduction in the risk of death. But the subgroup meta-analysis concerning gemcitabine and docetaxel failed to show positive benefits in PPC. Although the association between histology and survival in NSCLC is controversial [7], [9], [19], our results show a significant 13% OS improvement in non-squamous patients treated with pemetrexed. One potential explanation is that higher TS gene expression in squamous cell carcinoma compared with adenocarcinoma may confer relative resistance to pemetrexed [20], [21]. There were more non-squamous patients than squamous patients in the selected four trials (from 70% to 100%), implying that non-squamous patients might play a greater role in the meta-analysis of OS for all NSCLC patients. Scagliotti et al. [7] reported reversed results in those with squamous histology treated with PP (HR = 1.22, 95% CI: 0.99–1.50, *p* = 0.05). However, there are not enough data to perform the meta-analysis of patients with squamous histology.

Regarding grade 3–4 toxicity data, our pooled analysis showed that pemetrexed produced less neutropenia and leukopenia, but more nausea. Subgroup analysis showed that pemetrexed produced more thrombocytopenia and anemia compared with docetaxel and less thrombocytopenia compared with gemcitabine.

To the best of our knowledge, this is the first meta-analysis to evaluate the efficacy and safety of PPC as the first-line chemotherapy in patients with advanced NSCLC. Several non-random phase II trials showed that pemetrexed plus platinum regimens were associated with median survival times of 8.9 to 13.5 months and RRs of 24% to 45.8% [22], [23], [24] However, comparing with other PBR, our results of four RCTs demonstrated that pemetrexed regimens could improve OS in advanced NSCLC patients, especially in non-squamous NSCLC patients, but not PFS and RR, and PPC was well tolerated with less neutropenia and leukopenia but more nausea. Recently, a combined analysis by Treat et al. [25] also showed that PP was associated with favorable survival when compared with GP in non-squamous NSCLC patients but not in all patients.

There were several limitations in our study. First, as there were only four RCTs and some data were not reported, these results need to be interpreted very cautiously. As only non-squamous data were available for analysis while other data such as HRs for OS based on squamous histology, gender and age were not mentioned in most studies, further analysis of individual patient data is needed to confirm our findings. Second, although publication bias was not found according to funnel plots and Egger's test, the small number of trials and possible existence of unpublished studies limited the power of these tests. Furthermore, the method used to calculate HRs and different covariates used for HRs adjustment may lead to potential bias. We calculated HR, log HR, and its variance from the data or the survival curves included in the article. In addition, HRs in the studies were adjusted for different covariates, and covariates were not consistent even in multivariate analysis performed in different studies. Third, because this study is based on the trials of gemcitabine and docetaxel, the results are not necessarily applicable to treatments that use other drugs. Subgroup analysis showed negative results for OS in gemcitabine-based trials. Fourth, our results were pooled from four RCTs of chemotherapy in advanced NSCLC. However, erlotinib conferred a significant progression-free survival benefit in patients with advanced EGFR mutation-positive NSCLC [26]. Although mutational profiling has not yet been widely adopted into practice, customizing targeted therapies to specific mutations may be more effective for some types of cancer [27]. Therefore, the conclusions should be applied to patients unsuitable for targeted therapy. Finally, our results were inconsistent with other studies. Treat et al. [25] also reported that no significant benefit for OS was observed in all patients or non-squamous patients treated with PP, when compared with GC, gemcitabine/paclitaxel, and paclitaxel/carboplatin. Therefore, more trials comparing PPC with PBR are needed to evaluate the efficacy of pemetrexed in chemotherapy-naive advanced non-squamous NSCLC patients.

In conclusion, this meta-analysis demonstrates that PPC in the first-line setting leads to a significant survival advantage for advanced NSCLC patients and non-squamous patients compared with other PBR. Taking into account less toxicity (such as neutropenia and leukopenia), PPC could be considered as the first-line treatment option for patients with advanced NSCLC, especially those with non-squamous histology.

## Supporting Information

Figure S1 **The flow of the included studies.**
(DOC)

Checklist S1 **PRISMA checklist.**
(DOC)
