# Supplementary material for: Pemetrexed plus Platinum as the First-Line Treatment Option for Advanced Non-Small Cell Lung Cancer: A Meta-Analysis of Randomized Controlled Trials
Source: PLoS One. 2012 May 17;7(5):e37229. doi: 10.1371/journal.pone.0037229 (PMC3355109; doi:10.1371/journal.pone.0037229)
Supplement: Figure S1 — The flow of the included studies. (DOC) [file pone.0037229.s001.doc]

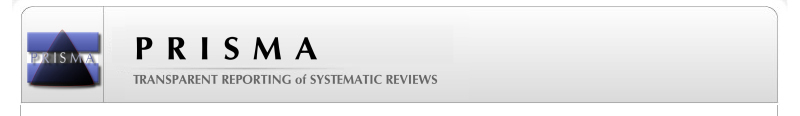
**PRISMA 2009 Flow Diagram**

**Screening**

**Included**

**Eligibility**

**Identification**

Records identified through database searching
(n = 702 )

Additional records identified through other sources
(n =131 )

Records after duplicates removed
(n = 803 )

Records screened
(n =122)

Records excluded
(n =102 )

Full-text articles assessed for eligibility
(n =20)

Full-text articles excluded, with reasons
(n =7)

Studies included in qualitative synthesis
(n =13 )

Studies included in quantitative synthesis (meta-analysis)
(n =4)
